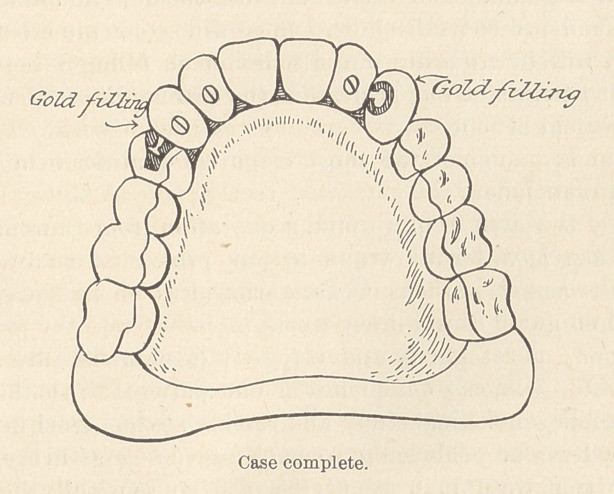# Removable Porcelain Crown- and Bridge-Work

**Published:** 1897-05

**Authors:** Adam Flickinger

**Affiliations:** St. Louis, Mo.


### REMOVABLE PORCELAIN CROWN- AND BRIDGE-WORK.[1]

BY ADAM FLICKINGER, D.D.S., ST. LOUIS, MO.

FROM the earliest history to the present day the dental profession has recognized the fact that artificial teeth, mounted on base plates, of whatsoever kind, have not fully met the approbation of all concerned. While gold, platinum, silver, rubber, celluloid, tin, aluminum, and other metals and compositions have been and are still in use and have their merits, the profession acknowledges, in spite of science and skill, that prosthetic dentistry has made but little progress in the method of constructing a better, more comfortable denture, and one more in harmony with the original conditions of the oral cavity.

In crown- and bridge-work we have the ideal mechanism with which to restore the lost or sacrificed members, without adding a surplus of material, or covering such portions of living tissue which by nature were not destined to be encased and buried forever. This class of work has demanded the attention of the profession for many years, and many methods and appliances have been suggested and advocated from time to time by some of the most able men following our vocation. Among them we find Drs. Webb, H. C. Register, Finley Thompson, J. N. Williams, R. Walter Starr, James B. Hodgkin, G. W. Mellote, Wilbur F. Litch, W. Storer How, E. Parmley Brown, Bonwill, Sidney Stowell, C. M. Richmond, James W. Low, J. L. Davenport, George Evans, W. N. Morrison, and J. R. Callahan.

Dr. Webb's method, described in a paper entitled "Grafting Crowns in Lieu of Plates," which was read before an eastern dental society a quarter of a century ago and brought forth so much discussion at the time, has been almost forgotten.

Dr. Bing's method of bridge-work (retained by fillings), patented, if I am not mistaken, received considerable attention, and of recent

---

[1] Read before the New York Institute of Stomatology, February, 1897.

date, Dr. Condit's method of "removable bridge plates" is advocated by some, and illustrated in different dental periodicals to a great extent.

Still, after all that has been written and published heretofore, the principle of bridge-work may yet be considered in its infancy; and the profession has apparently made but little progress towards the adoption of any one method which meets the general approval and satisfactorily solves the problem.

Cheap dentistry is, no doubt, one of the causes of the lethargy observed in dental prosthetics, for it must be admitted by all who study the subject that vulcanite, more than any other class of manipulatory work, has been the means of lowering the standard of prosthesis. The ease with which a vulcanite plate can be constructed, the little skill required in learning to flask, vulcanize, and finish a rubber set of teeth (aside from taking the impression and arranging the articulation), has opened an attractive field for a class of practitioners whose sole aim on entering the "business," as they term it, is to make an easy living, by doing cheap work and much of it; in this way they come into competition with the more learned and skilful, who in turn are obliged to let prosthetic work go and turn their attention and knowledge towards operative and medicinal dentistry. Vulcanite work, as one prominent St. Louis dentist said, "is a boon to the poor people," but, unfortunately, it has created a demand for cheap dentistry even among people who can well afford to pay for a higher grade of work.

If hearers and readers will observe the illustrations, and follow the description of a system of removable porcelain crown- and bridge-work herewith presented, they will find that it possesses many advantages over other methods of bridge-work and artificial dentures in general use, avoiding the mutilation of teeth and an undue display of metal.

Being constructed of a heavy iridio-platinum saddle, onto which the teeth are soldered and baked, the whole fits snugly over a heavy bar of the same metal, resting firmly on the gum, and is anchored to natural teeth, roots or crowns, assuring perfect security against absorption of fluids and lodgement of food. The metal used does not invite or cause inflammatory conditions so frequently met with under bridge-work constructed of gold; at the same time its tenacity insures proper strength.

The model of Case V., exhibited to-night, shows five anterior teeth. It was illustrated and described in the May number of the *Dental Review*, 1896, and is a duplicate of a case made over four years ago for a young man who had the misfortune to lose his teeth in a runaway accident, in which his nasal, malar, and maxillary bones and several teeth were badly fractured. Considerable surgical treatment was required before the construction of this bridge-work, which latter has given entire satisfaction so far, with every indication that it will continue to do so for years to come.

The illustration, Case IX., showing a single, removable crown and its various extensions, is an interesting study, and is copied from practical cases.

CASE IX.

Case IX. shows a single crown and extension. *C'*, crown; *A*, *A*, root and pier; *B*, bar in position; *C*, *D*, teeth soldered on saddle; *E*, finished.

Case XIII. shows illustrations, models, and photos. of a case with combination anchorages. Examination of plaster model will show that the upper incisors project considerably beyond the lower teeth, with the right canine inside of the arch. The teeth, being badly decayed and containing dead pulps, did not, in my estimation, justify treatment, regulation, and subsequent filling; therefore I concluded to extract the two centrals and right canine. The lateral roots were utilized to assist in carrying the bridge-work. By comparing models may be seen the change brought forth by a temporary plate replacing the extracted teeth, made to allow time for shrinkage of the gum and for moving the lateral roots mesially and inwardly, thereby reducing the enormous space the centrals had previously occupied, at the same time gaining room for the canine.

The sketches of this work, Case XIII., illustrate the principle of binding the pier to an abutment. On the left the lateral root is bound to the canine, on the right the lateral root to the bicuspid by a girder-bar stretching across the canine space. Both bars are retained by ferruled posts in the roots and gold fillings in the teeth.

It will be noticed that my system differs materially from Dr. E. Parmley Brown's, inasmuch as his girder-bar and porcelains are all connected, while my porcelains are easily removed individually from the bar, allowing replacement in case of accident.

In conclusion, allow me to thank you for the interest shown in this system of removable porcelain crown- and bridge-work.

Case XIII.

*Saddle in position*

Admitting that I do not consider this or any other form, system, or method of bridge-work a cure for all toothless people, I claim

Case complete.

that if it be carried out with due regard to the fundamental requirements,—namely, good anchorage, piers, abutments, etc.,—it is a blessing to many patients, and a memorial to the operator more lasting than the golden monuments carried about in the mouths of many of our fellow-beings, advertising the fact that prosthetic dentistry has rather retrogressed in comparison with other branches and studies in dentistry.

We should bear in mind, as Bacon says, that our studies should be neither "a couch on which to rest; nor a cloister in which to promenade alone; nor a tower from which to look down on others; nor a fortress whence we may resist them; nor a workshop for gain and merchandise; but a rich armory and treasury for the glory of the Creator and the ennoblement of life."

## Figures and Tables

**Case IX. f1:**
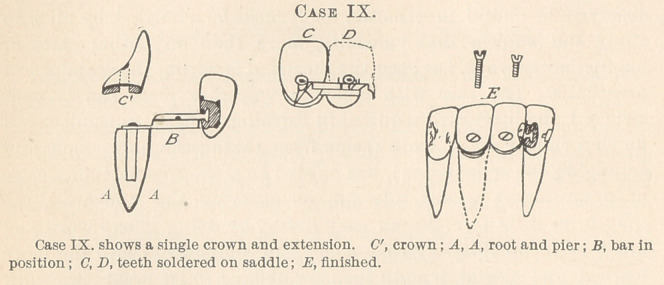


**Case XIII. f2:**
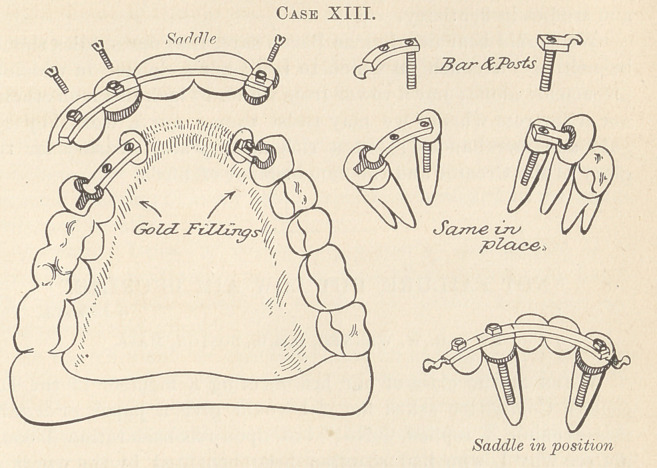


**Figure f3:**